# Clinicopathologic features and genomic profiling of female axillary lymph node metastases from adenocarcinoma or poorly differentiated carcinoma of unknown primary

**DOI:** 10.1007/s00432-024-05783-6

**Published:** 2024-05-15

**Authors:** Liansha Tang, Yueting Zhu, Yang Du, Xiangyu Long, Yixiu Long, Yuan Tang, Jiyan Liu

**Affiliations:** 1 Department of Biotherapy, Cancer Center, West China Hospital of Sichuan University, 37 Guoxue Xiang Street, Chengdu, 610041 Sichuan Province China; 2 Biotherapy Clinical Research Center of Sichuan Province, Chengdu, 610041 China; 3 Department of Gynecological Oncology, Fudan University Shanghai Cancer Center, Fudan University, Shanghai, 200032 China; 4 Department of Oncology, Shanghai Medical College, Fudan University, 270 Dong’an Road, Shanghai, 200032 China; 5 Department of Pathology, West China Hospital of Sichuan University, Chengdu, 610041 China

**Keywords:** Axillary lymph node metastases, Cancer of unknown primary, CUPAx, Genomic profiling, Occult breast cancer

## Abstract

**Purpose:**

Axillary lymph node metastases from adenocarcinoma or poorly differentiated carcinoma of unknown primary (CUPAx) is a rare disease in women. This retrospective study intended to examine the clinicopathological features of CUPAx and compared CUPAx genetically with axillary lymph node metastases from breast cancer (BCAx), investigating differences in their biological behavior.

**Methods:**

We conducted the clinical and prognostic analysis of 58 CUPAx patients in West China Hospital spanning from 2009 to 2021. Gemonic profiling of 12 CUPAx patients and 16 BCAx patients was conducted by the FoundationOne CDx (F1CDx) platform. Moreover, we also compared the gene mutation spectrum and relevant pathways between the two groups and both TCGA and COSMIC databases.

**Results:**

The majority of the 58 CUPAx patients were HR-/HER2- subtype. Most patients received mastectomy combined radiotherapy (50 Gy/25f). CUPAx patients who received mastectomy instead of breast-conserving surgery had a more favorable overall prognosis. Radiotherapy in chest wall/breast and supraclavicular/infraclavicular fossa was the independent prognostic factor (HR = 0.05, 95%CI = 0.00–0.93, P = 0.04). In 28 sequencing samples (CUPAx, n = 12, BCAx, n = 16) and 401 TCGA-BRCA patients, IRS2 only mutated in CUPAx (33.33%) but amplified in BCAx (11.11%) and TCGA-BRCA (1.5%). Pathway analysis revealed that BCAx had more NOTCH pathway mutations than CUPAx. Enrichment analysis showed that CUPAx enriched more in mammary development and PML bodies than BCAx, but less in the positive regulation of kinase activity.

**Conclusions:**

More active treatment methods, like chemotherapy, mastectomy and postoperative radiotherapy, could improve the prognosis of CUPAx. The differential mutation genes of CUPAx and BCAx might be associated with their respective biological behaviors like invasiveness and prognosis.

**Supplementary Information:**

The online version contains supplementary material available at 10.1007/s00432-024-05783-6.

## Introduction

Cancer of unknown primary site (CUP) is a heterogeneous disease diagnosed histologically as a metastatic tumor, for which the primary site remains undefinable even after standardized diagnostic workup and detailed examination. Recent data shows CUP accounts for 1.7% and 7.9% of estimated new cancer cases and deaths in the United States in 2023 (Siegel et al. 2023), respectively. Approximately 30–40% of CUP cases occur in lymph nodes (Hayashi et al. 2019; Qi et al. 2023). More than 80% of axillary masses are lymphadenopathies, of which more than 90% of isolated axillary lymph node metastases are pathologically diagnosed as poorly differentiated carcinoma or metastatic adenocarcinoma.

Axillary lymph node metastasis from adenocarcinoma or poorly differentiated carcinoma of unknown primary (CUPAx) is a subtype of CUP (Pentheroudakis et al. 2010). The most common secondary malignant tumors in the axilla are orignated from the lung, breast, thyroid, gastrointestinal tract, ovary, and uterus. Since nearly 75% of breast lymphatic drainage flows to the axillary lymph nodes, the breast is considered the most common primary site. Especially for female patients, if they present with isolated axillary lymph nodes metastasis (adenocarcinoma or poorly differentiated carcinoma), breast cancer should be suspected. This subgroup shares similar biological characteristics with stage II-III breast cancer and is named occult breast cancer (OBC).

CUPAx is extremely rare. Current understanding is mainly based on case reports and small-scale clinical studies. The clinicopathologic and prognostic features are still obscure. Previous literature indicated that CUP patients suffered a poorer prognosis than those with metastatic tumors from known primary sites (Rassy et al. 2020; Jackson et al. 1995). While some studies from large databases suggested patients with CUPAx had more prolonged overall survival (OS) and breast cancer-specific survival (BCSS) than breast cancer with axillary lymph node metastasis (BCAx) (Huang et al. 2020; Zhao et al. 2022). In addition, there is no consensus on the treatment of CUPAx, especially in the management (mastectomy or breast-conserving surgery (BCS)) of the breast without the primary lesions. Growing evidence demonstrated no significant differences between these two methods in improving prognosis (Macedo et al. 2016; Sohn et al. 2014; McCartan et al. 2017). In recent years, with the advancement of genomics analysis, a deep understanding of the genetic background and molecular characteristics of cancer was gained. Gene mutation analysis played a driving role in tumor precision targeted therapy. Currently, there is no relevant analysis of gene mutations in CUPAx. Potential targeted therapies are still warranted to be explored and summarized.

Accordingly, our study intended to explore the clinical and prognostic characteristics of CUPAx. We also conducted a comprehensive genomic profiling analysis in CUPAx and compared it with BCAx to find the differences in biological behavior between them, providing a reference for treatment guidance.

## Material and methods

### Patient inclusion

We extracted the clinical and pathological data of metastatic axillary lymph nodes of unknown primary from 2009 to 2021 in West China Hospital. Among them, female cases diagnosed with adenocarcinoma or poorly differentiated carcinoma in the axilla, with no distant metastasis at initial diagnosis, and no primary lesion found by comprehensive clinical and imaging examinations (mammography, ultrasound, CT etc.) were classified as CUPAx (clinical type). Cases with negative MRI or undergoing breast surgery without lesions identified in postoperative pathology were defined as CUPAx (pathological type). Both clinical and pathological types of CUPAx were recruited in the subsequent clinical and prognostic analyses. Patients with previous cancer history, no pathological diagnosis, and tissue morphology and immunohistochemistry indicating non-breast origin were excluded from this study. Baseline features, therapy information and survival data were extracted from the medical record. The positive axillary lymph node ratio is determined by dividing the number of axillary nodes containing cancer by the total number of axillary nodes examined. The definition of pathological complete response (pCR) was that no positive lesions were found in the axillary pathology examination following neoadjuvant chemotherapy. For patients who underwent excisional biopsy before neoadjuvant chemotherapy and did not undergo subsequent pathological examination, they were classified as "Unknown" in axillary downstaging.

The “Masked Somatic Mutation”, “Masked Copy Number Segment”, and clinical data of BCAx (T1-3N1-3M0) were selected from The Cancer Genome Atlas (TCGA) database (TCGA-BRCA) (https://portal.gdc.cancer.gov/). VarScan software was utilized to preprocess somatic mutation data, which was visualized by the R package “maftools”. The deletion and amplification of copy number variations (CNV) were reflected by “Segment_mean” values, where less than 0 was deletion, and more than 0 was amplification. Clinical variables included age, estrogen receptor (ER)/progesterone receptor (PR)/human epidermal growth factor receptor 2 (HER2) status, TNM stage. Data with incomplete survival and TNM stage were excluded.

### Study endpoints

The primary data endpoints in West China Hospital were OS and disease-free survival (DFS). OS and DFS were defined as the time from treatment to death or tumor recurrence. For patients with no recurrence, DFS was calculated as the interval between the last follow-up date or death date from the start of treatment.

### Comprehensive genomic profiling

Formalin-fixed paraffin-embedded (FFPE) biopsy/excision/surgical specimens of axillary lymph nodes from CUPAx and BCAx (T1-3N1-3M0) were obtained spanning from 2016 to 2021 in West China Hospital. FoundationOne CDx (F1CDx) platform, applied by DIAN (Hangzhou Lab) with licensed technology, was conducted to sequence samples. This gene panel assay has been approved by FDA, and the sequence methods have also been validated and described before (Frampton et al. 2013). A total of 324 gene alterations were detected, including short variants (base substitutions, insertions and deletions (INDELs)), and structural variants (gene rearrangements and copy number changes). Genomic features, such as tumor mutation burden (TMB) and microsatellite instability (MSI), were also reported by F1CDx.

### Statistical analysis

T-test or Wilcoxon rank-sum test or sign-rank test would be used to analyze continuous variables. The Kaplan–Meier analyses and log-rank test were performed to describe survival rates and compare survival differences. Univariate and multivariate Cox proportional hazards regression were conducted to analyze prognostic factors, calculated by hazard ratios (HRs) and 95% confidence intervals (CIs). All statistical analysis were two-sided. P-value < 0.05 indicated statistical significance.

The statistical analysis of gene mutations was based on dichotomy (alteration or not). Chi-square test and Fisher's exact test were performed to determine the discrepancies in TMB and mutations/pathway alterations between two groups. The maftools package in R software was assessed the mutual exclusion and co-occurrence of candidate genes. GO and KEGG analysis were implemented for functional and pathway analysis of target genes by the "clusterProfiler" package in R software. All statistical analysis were performed using R software version 4.2.2. This study has been approved by the ethics committee of West China Hospital (Reference number: 2022 Num.846, Aug 23rd, 2022).

## Results

### The clinicopathological features of CUPAx

We recruited 58 eligible CUPAx patients in West China Hospital from 2009 to 2021. The inclusion and exclusion flow charts were shown in Supplementary Fig. S1. The pathological subtypes were mainly invasive carcinoma of no special type (43.10%) and poorly differentiated carcinoma (12.07%) (Table S1). About 25% (15/58) of CUPAx patients presented suspicious breast lesions and received breast biopsy, but no positive results were found. Primary lesions were found in 6 patients (clinical CUPAx) during subsequent treatment between two years and six years. The histological types of found breast lesions mainly were ductal carcinoma in situ. In CUPAx patients, less than 55 years old, stage N1, metastatic lymph nodes first occurred in the left axilla, HR-/HER2- subtype accounted for the largest part.

**Fig. 1:**
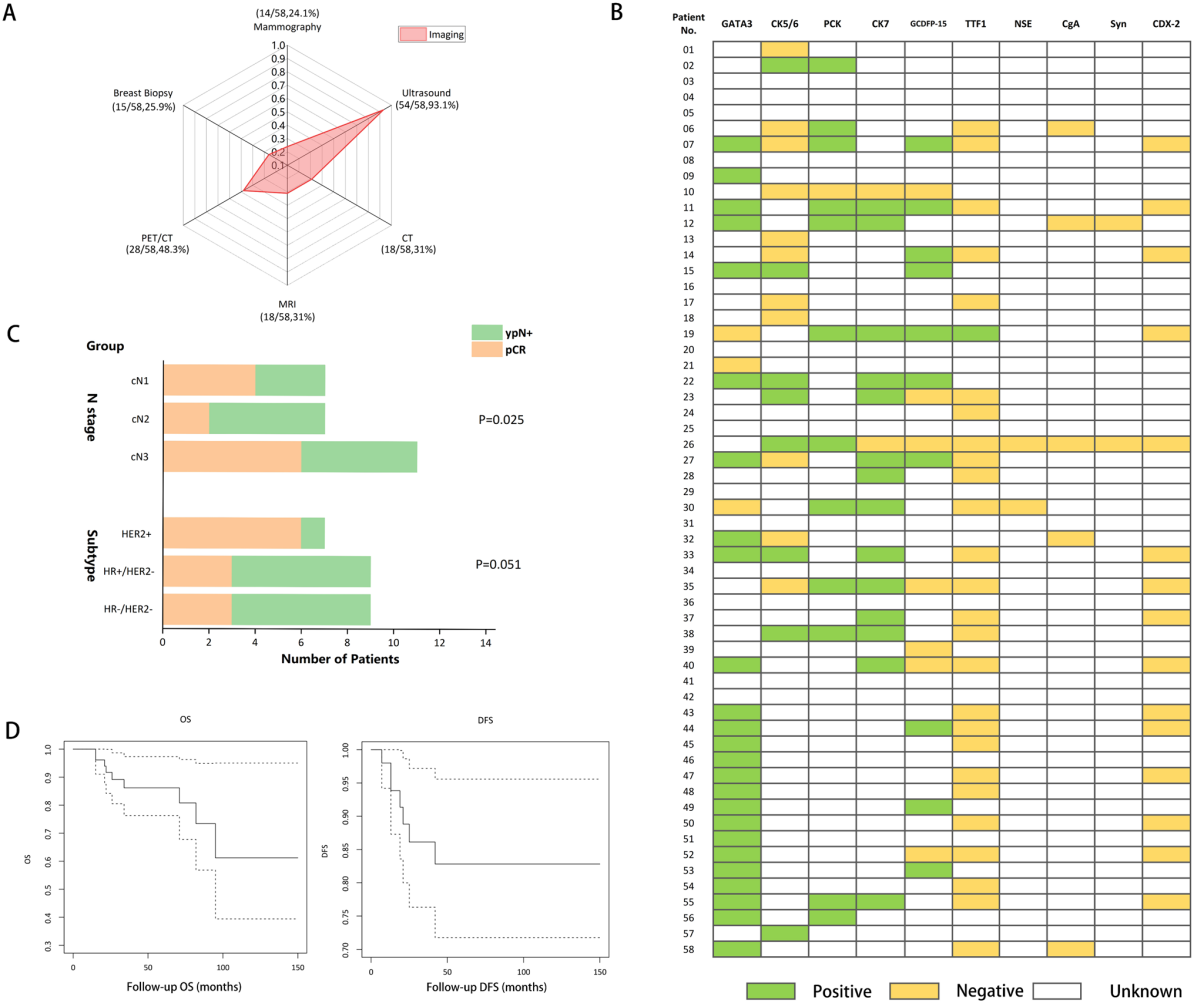
**A** The imaging examinations of 58 CUPAx patients. **B** The immunohistochemical markers of 58 CUPAx patients. **C** The axillary downstaging of neoadjuvant chemotherapy in CUPAx patients stratified by N stage and subtypes. **D** The OS and DFS of CUPAx patients

The imaging examinations included ultrasound, mammography, CT, MRI and PET/CT (Fig. 1A). Among them, ultrasound (93%) and PET/CT (48%) were the predominant examination methods. Immunohistochemical markers included breast-related markers (GATA3, GCDFP15, CK5/6), epithelial markers (CK7, PCK), neuroendocrine-related markers (NSE, CgA, Syn), lung-related markers (TTF-1), and intestinal-related markers (CDX-2) (Fig. 1B). Among them, the highest numbers of tumor samples expressed GATA3, CK7, PCK, CK5, GCDFP15 were 25 (43%), 21 (36%), 19 (33%), 13 (22%), and 10 (17%) respectively.

### The multiple treatments of CUPAx

The therapies included surgery, radiotherapy, chemotherapy, endocrine therapy, and targeted therapy (Supplementary Table S1). Over 60% of patients underwent breast mastectomy, while only eight patients received BCS. As for the axillary surgery, 66% of patients underwent axillary lymph node dissection (ALND), followed by about 20% of patients who only received axillary lymph node sampling (ALNS), and the rest underwent axillary mass excision. The vast majority of patients received radiotherapy and chemotherapy. The most common comprehensive treatment for patients was breast mastectomy combined with radiotherapy (Table 1). 27 of 34 patients who received radiotherapy had definite information about the location and dose. The locations of radiotherapy included chest wall/breast, axilla, supraclavicular and infraclavicular fossa (SCF/IVF). Among them, chest wall/breast + SCF/IVF took the largest part (77.78%, 21/27). Five patients received axillary radiotherapy. The radiological dose was 50 Gy/25f, and three patients considered tumor bed boost. Nearly 50% of CUPAx patients received neoadjuvant chemotherapy, and about 20% of patients achieved pCR after treatment, while 22% of patients remained pathologically node positive (yN +) in the axillary lymph nodes (Supplementary Table S1). A more significant decrease in the axillary stage was found in patients with N3 and HER2 + subtypes (Fig. 1C). More than 60% of patients received adjuvant chemotherapy, which primarily consisted of anthracyclines, taxanes, and platinum-based regimens. Nearly 30% of patients received endocrine therapy or targeted therapy.

**Table 1:** Type of radiotherapy according to surgical therapy and clinical N status of CUPAx

Radiotherapy sites	Chest wall/breast + SCF/IVF	Chest wall/breast + axilla + SCF/IVF	Axilla + SCF/IVF	SCF/IVF	P-value
Number	21	4	1	1	
Dose					0.02
50 Gy/25f	20 (95.24%)	3 (75.00%)	0 (0.00%)	1 (100.00%)	
50 Gy/25f + tumor bed boost	1 (4.76%)	1 (25.00%)	1 (100.00%)	0 (0.00%)	
N stage					0.27
cN1	11 (52.38%)	0 (0.00%)	0 (0.00%)	1 (100.00%)	
cN2	3 (14.29%)	2 (50.00%)	0 (0.00%)	0 (0.00%)	
cN3	7 (33.33%)	2 (50.00%)	1 (100.00%)	0 (0.00%)	
Surgery					< 0.001
Mastectomy	19 (90.48%)	3 (75.00%)	0 (0.00%)	0 (0.00%)	
BCS	1 (4.76%)	0 (0.00%)	0 (0.00%)	0 (0.00%)	
ALND	0 (0.00%)	0 (0.00%)	0 (0.00%)	1 (100.00%)	
ALNS	1 (4.76%)	1 (25.00%)	1 (100.00%)	0 (0.00%)	
Breast operation					0.02
No	2 (9.52%)	1 (25.00%)	1(100.00%)	1 (100.00%)	
Mastectomy	19 (90.48%)	3 (75.00%)	0 (0.00%)	0 (0.00%)	
Axillary Operation					0.13
ALND	19 (90.48%)	3 (75.00%)	0 (0.00%)	1 (100.00%)	
Excision	1 (4.76%)	0 (0.00%)	0 (0.00%)	0 (0.00%)	
ALNS	1 (4.76%)	1 (25.00%)	1 (100.00%)	0 (0.00%)	

*SCF/IVF* supraclavicular and infraclavicular fossa, *BCS* breast-conserving surgery, *ALND* axillary lymph node dissection, *ALNS* axillary lymph node sampling. ALND in Surgery means patients only received ALND, while in Axillary operation focuses on axillary, it also includes Mastectomy + ALND

### The prognosis of CUPAx

**Fig.2:**
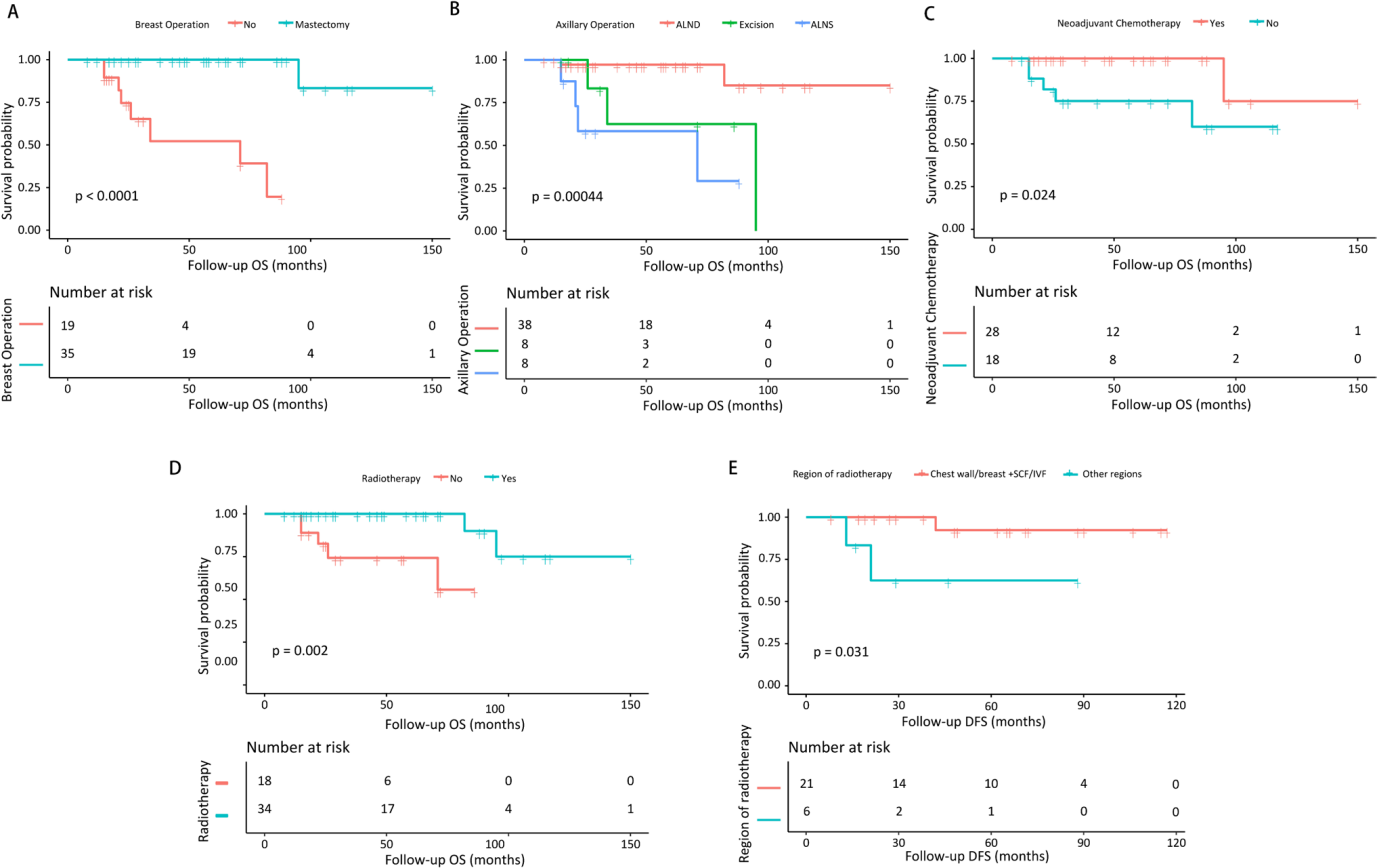
The K-M curves of OS and DFS in CUPAx patients. **A** Breast operation and OS, **B** Axillary operation and OS, **C** Neoadjuvant chemotherapy and OS, **D** Radiotherapy and OS, **(E)** Region of radiotherapy and DFS. OSM: overall survival months; DFSM: disease-free survival months

54 patients had complete follow-up information, with an average follow-up time of 50.13 months. The overall prognosis of CUPAx was favorable. The 10-year OS and DFS was 60% and 83% respectively (Fig. 1D). The K-M curve (Fig. 2) showed that patients who received radiotherapy, breast mastectomy, ALND, and received neoadjuvant chemotherapy had a superior OS (P < 0.05). Among the total of 58 patients, seven experienced disease progression within six months to three years after treatment. Of the 28 patients who underwent radiotherapy (with specified treatment sites and doses), three developed distant metastases, comprising two cases with lung and bone metastases respectively, and one with metastasis to the anterior superior mediastinal lymph nodes. Notably, none of these metastases occurred at the sites previously irradiated. Among the remaining 30 patients, 4 encountered local recurrence, manifested by axillary recurrence and SCF/IVF lymph node metastasis. Patients with chest wall/breast + SCF/IVF radiotherapy showed fewer cases of recurrence/progression than all other regions of radiotherapy (P = 0.03) (Fig. 2E). In addition, univariate analysis (Supplementary Table S2) showed that radiotherapy, ALND, and the positive axillary lymph node ratio less than 25% were the significant prognostic factors for OS. Radiation fields limited to the SCF/IVF regions alone did not significantly control disease progression. Multivariate analysis (Supplementary Table S3) showed that radiotherapy was an independent prognostic factor for OS (HR = 0.05, 95% CI = 0.00–0.93, P = 0.04).

### The comparison of baseline characteristics between CUPAx and BCAx

We finally recruited 28 samples that qualified for gene sequencing. Of these, 12 samples were in the CUPAx group and 16 samples were in the BCAx group (Supplementary Table S4). All BCAx patients underwent mastectomy, while half of the CUPAx patients did not. The rates of pCR (58.33% vs. 18.75%, P = 0.04) and HER2- subtype (83.33% vs. 37.50%, P = 0.02) in the CUPAx group were significantly higher than those in the BCAx group.

### The genomic profiling of CUPAx and BCAx

All 28 patients had 214 gene mutations, averaging of 7.6 gene mutations per sample. The clinically relevant gene mutations were shown in Supplementary Table S5. The top 3 target gene mutations were PIK3CA (39.13%), ERBB2 (39.13%), and BRCA1/2 (21.74%). The maximum TMB of the CUPAx and BCAx group was 30Mut/Mb and 11Mut/Mb respectively. All patients were MSS. The TCGA database included 401 T1-3N1-3M0 breast cancer patients, with 11,182 gene alterations and 24,219 CNVs. The range of TMB was 0.06–12.36 Mut/Mb.

**Fig. 3:**
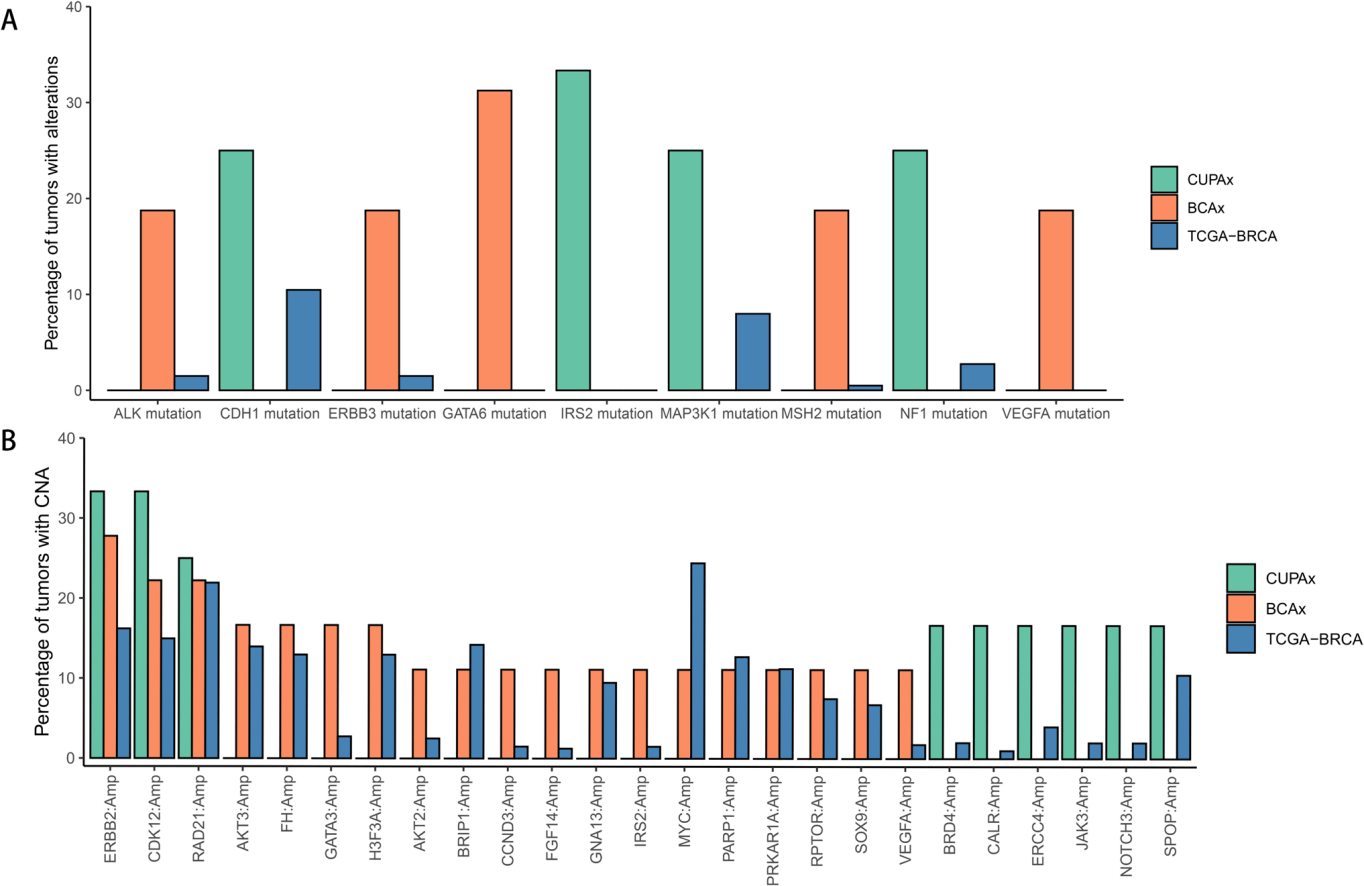
The comparison of short variants (**A**), copy number variations (CNVs) (**B**) among 12 CUPAx, 16 BCAx, and 401 TCGA-BRCA patients

The frequency and types of gene mutations were shown in Supplementary Fig. 2. The results of the three groups stratified by subtypes were shown in Supplementary Fig. S3A. The differences among the three subtype groups in three groups were not significant. The top three mutated genes (TP53, CDK12, ERBB2) still showed no significant differences between CUPAx and BCAx groups (Supplementary Fig. S3B). We selected the genes that mutated in more than three patients in either group, compared with the gene frequencies in the TCGA database (Fig. 3). As for the short variations, we found that IRS2 had short variations only in the CUPAx group, while GATA6 mutated only in the BCAx group. Regarding the CNV, GATA3 and IRS2 did not show amplification in the CUPAx group. Specifically, GATA6, IRS2, and VEGFA had mutations in the CUPAx or BCAx group, but no mutations were found in the TCGA database. Therefore, we investigated these genes in the COSMIC database (https://cancer.sanger.ac.uk/cosmic) of breast cancer. The results showed that all three genes had specific mutation frequencies (GATA6: 0.7%, IRS2: 1.1%, VEGFA: 0.7%).

The co-mutated genes analysis in the CUPAx and BCAx groups was shown in Supplementary Fig. S4A. MAP3K1, BRIP1 and PIK3CA showed significant co-occurrences of mutations in the CUPAx group, while the BCAx group found significant co-occurrences of mutations in PIK3R1 with ROS1 and AXIN1. We also described the mutation sites of IRS2 and GATA6 (Supplementary Fig. S4B). The mutation sites of IRS2 in the CUPAx group were A512T (2/16), C97Y (1/16), and R558P (1/16). Compared with the COSMIC database, C97Y was a new site. All the GATA6 mutation sites, G244S (2/16), D5E (1/16), S184N (1/16), and H331_H333del (1/16), were new sites, which were not found in the COSMIC database.

### The pathway analysis of CUPAx and BCAx

**Fig. 4:**
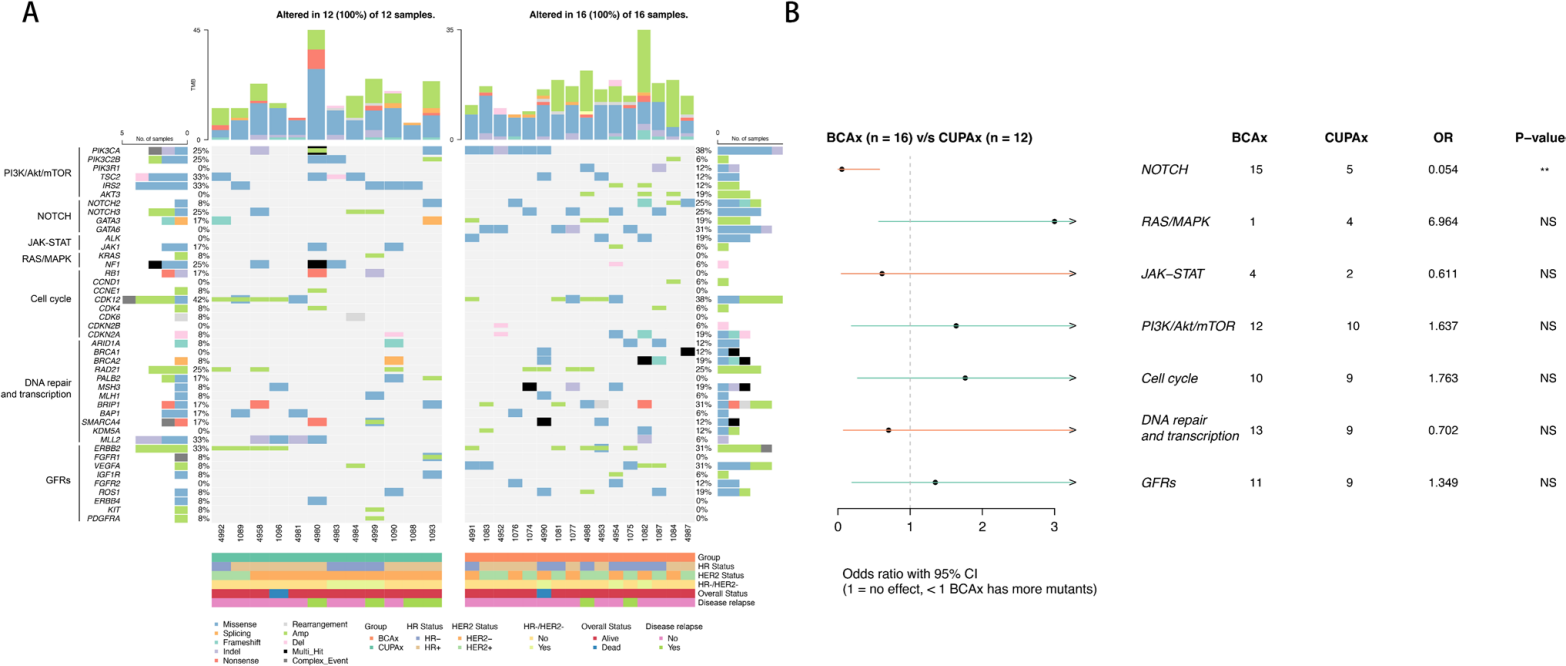
The mutation gene stratified by pathways (**A**) and pathway differences (**B**) between 12 CUPAx and 16 BCAx patients

**Fig. 5:**
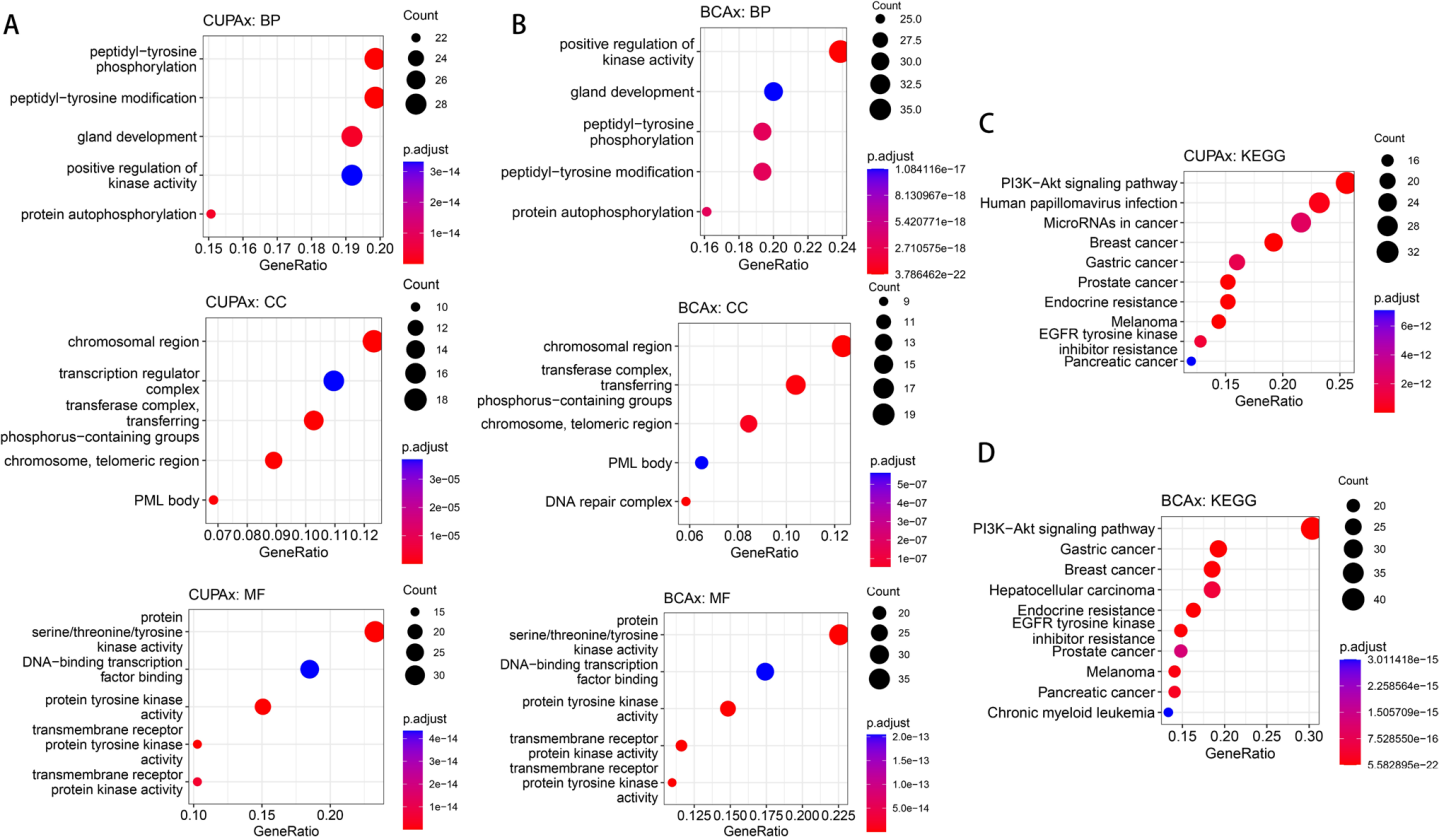
GO and KEGG pathway enrichment analysis of 12 CUPAx (**A**, **C**) and 16 BCAx patients (**B**, **D**). The top 5 enriched GO (**A**, **B**) and 10 enriched KEGG (**C**, **D**) pathways were listed. The bubble size typically represented the number of genes associated with the pathway. The bubble color indicated significance level, with dark red indicating a lower p-value and higher significance, and blue indicating the opposite. The GO analysis showed that the CUPAx group had a higher enrichment in gland development and PML bodies pathway, but a lower enrichment in positively regulating kinase activity than BCAx group. The KEGG analysis did not found differences between these two groups. *GO* gene ontology; *KEGG* kyoto encyclopedia of genes and genomes; *BP* biological process; *MF*, molecular function; *CC* cellular component

We compared the mutated genes in the CUPAx and BCAx groups according to pathway classification (Fig. 4A). The differences in pathway mutations between CUPAx and BCAx were presented in Fig. 4B. NOTCH pathway mutations showed more relevant gene mutations in the BCAx group (P < 0.05) than the CUPAx group. We conducted a correlation analysis of pathway mutations with molecular subtypes and disease prognosis, but no significant relationship was found between them (data not shown). The GO and KEGG analysis of BCAx and CUPAx groups were shown in Fig. 5. The GO analysis showed that the CUPAx group had a higher enrichment in gland development and promyelocytic leukemia protein (PML) bodies pathway, but a lower enrichment in positively regulating kinase activity. The KEGG analysis showed that the PI3K/Akt/mTOR pathway was the most enriched in both groups.

## Discussion

### The clinical and pathological features of CUPAx

Female patients with CUPAx exhibit characteristics resembling stage II-III breast cancer and are commonly referred to as OBC. Recent literature proposed the concepts of clinical OBC (cOBC) and pathological OBC (pOBC) (Ofri et al. 2020). The former denoted the absence of breast lesions in physical examination, mammography, and ultrasound. The latter indicated negative breast MRI and postoperative pathological slices (with 5 mm intervals). In our study, more patients received PET/CT than MRI (28/58, 48.3% vs. 18/58, 31%). The above patients had negative PET/CT or breast MRI results. The sensitivity and specificity of PET/CT were both 84% in detecting occult lesions (Kwee et al. 2009), which was similar to MRI (96% and 63%, respectively) (Fayanju et al. 2013). Primary lesions were found in six CUPAx patients (cOBC), with four detected during breast surgery and the remaining two identified breast lesions two and six years after surgery. Therefore, most patients were pOBC in our study. We included patients with primary lesions found after surgery, as both types of patients were described in recent studies.

The immunohistochemical subtypes of CUPAx were diverse, but ER/PR status was pivotal for diagnosing breast origin. However, ER/PR negativity did not completely eliminate breast origin. The consistencies in ER/PR/HER2 status between primary breast tumors and metastatic lymph nodes ranged from 75.9 to 100% (Amir et al. 2012; Lindström et al. 2012), which was likely ascribed to differential expression in primary and metastatic sites. Since CUPAx lacked detectable primary lesions, the receptor status was detected through metastatic lymph nodes. False receptor detection results, tumor evolution and heterogeneity, could contribute to the inconsistencies mentioned above (Curtit et al. 2013; Pentheroudakis et al. 2007). Our results also showed that GCDFP-15 and GATA3 were highly expressed in CUPAx. GCDFP-15 was particularly sensitive to breast lobular carcinoma with high specificity (Raju et al. 1993), and GATA-3 was a marker for breast cancer but lacked specificity for CUPAx (Miettinen et al. 2014). Therefore, a more precise diagnosis of CUPAx requires the combination of multiple biomarkers.

### The treatment and prognostic factors of CUPAx

Currently, the main controversy surrounding CUPAx therapy is whether blind treatment should be performed on the breast without a primary lesion and which treatment methods should be used. Due to the psychological burden of unknown primary lesions on patients and the risk of recurrence and metastasis, observation only is currently not recommended (Foroudi et al. 2000).

Breast treatment options, mastectomy versus BCS, have been debated for years. BCS involved suspicious lesion excision + ALND + whole-breast radiotherapy (WBRT) or WBRT + ALND. Our study revealed that mastectomy was favored by the majority of CUPAx patients, consistent with other studies and the SEER database (Khandelwal et al. 2005; Fayanju et al. 2013). Blanchard et al. (2004) found that patients receiving mastectomy had better OS and DFS than non-mastectomy patients. Additional research indicated mastectomy had advantages over WBRT in reducing local recurrence (Foroudi et al. 2000; Ellerbroek et al. 1990; Campana et al. 1989). Our results presented that mastectomy patients had superior outcomes, possibly because 70% of them received neoadjuvant chemotherapy and 95% of them dissected axillary lymph nodes with postoperative radiotherapy. For non-mastectomy patients, half of them (7/14) did not undergo radiotherapy and ALND but received chemotherapy. Among the remaining patients who did not receive chemotherapy, three out of seven died. One of them underwent only axillary tumor excision, another underwent breast lump excision, and one refused treatment. These 14 patients were diagnosed between 2012 and 2017, and the reasons for not undergoing surgery were mostly based on patient preferences during follow-up. Similarly, prior study (Terada et al. 2022) have reported that more than half of CUPAx patients did not undergo mastectomy (55.6%) or breast radiotherapy (61.1%) from 2010 to 2014, but they all received chemotherapy (neoadjuvant or adjuvant), which was a favorable prognostic factor for CUPAx (Zhao et al. 2022). Perhaps from the patient's perspective, this could be the most suitable treatment for their economic condition based on comprehensive evaluation. Therefore, even though the breast lesions were covert, a favorable prognosis could be achieved when the management was equivalent to radical breast cancer treatment. Mastectomy has the most apparent benefit that possibly revealing evidence of primary lesions in the specimen, which were reported to appear from a few days to several years after diagnosis (He et al. 2012, Blanchard et al. 2004, Vlastos et al. 2001). Nonetheless, mastectomy could alleviate psychological stress of CUPAx patients.

Growing evidence suggested no significant differences in local recurrence, distant metastasis, and death rates between BCS and mastectomy (Walker et al. 2010; Macedo et al. 2016; Sohn et al. 2014; McCartan et al. 2017), no matter in patients with N1 or N2/N3 stage (Johnson et al. 2019). Recent clinical practice perspectives preferred WBRT for MRI-negative CUPAx patients. In our study, few patients received WBRT, and 90% of patients undergo chest wall radiotherapy after mastectomy. However, there is little evidence about chest wall radiotherapy (Masinghe et al. 2011; Rueth et al. 2015; Woo et al. 2013). McCartan et al. reported that 46% of patients received chest wall radiotherapy, but the recurrence of this subgroup was not described in detail (McCartan et al. 2017). In fact, WBRT might cause breast fibrosis and deformation, which contradicted the cosmetic goal of conserving the breast. According to the latest NCCN guidelines, MRI-negative patients need comprehensive evaluation on the basis of N stage. Patients with early-stage and no high-risk recurrence factors could choose mastectomy or BCS, whereas those with advanced stages were recommended to receive mastectomy after systemic treatment. Therefore, personalized decisions should be made for breast surgery, considering primary lesion detection, N stage, and patient preferences.

The combination of ALND and radiotherapy could effectively reduce recurrence and increase survival rates (Walker et al. 2010; Sohn et al. 2014; He et al. 2012). However, the optimal site and dose of radiotherapy for CUPAx were not widely reported. Regional lymph node irradiation like the SVF was commonly used in 70% of CUPAx patients (Barton et al. 2011). Our results showed all patients received local radiotherapy to the supraclavicular region, while only about 18% received axillary radiotherapy. Some literature did not clearly distinguish between axillary and supraclavicular irradiation, as they overlapped in some fields. For breast cancer, axillary radiotherapy was unnecessary if the ALND was thorough or the positive proportion was low. Therefore, for CUPAx patients without ALND, axillary radiotherapy was recommended. 50 Gy/25f was the commonly used dose, with no additional benefits in dose escalation from previous evidence (Barton et al. 2011).

### The differences of genomic profiling between CUPAx and BCAx

In recent years, few CUP cases including specific subgroups like CUPAx have been analyzed by genomic profiling (Binder et al. 2018; Ross et al. 2021). However, this is the first analysis of a separately reported CUPAX cohort to our knowledge. Notably, we found that CUPAx had more mutations in IRS2 compared to BCAx, and TCGA-BRCA did not have any IRS2 mutations, while BCAx had more IRS2 amplifications. IRS-2, a linking protein in the insulin-like growth factor 1 (IGF-1)/IGF-1 receptor (IGF-1R) pathway, mediates cell proliferation, migration, and survival by activating the PI3K/AKT/mTOR pathway (Mardilovich et al. 2009). Few studies described IRS2 mutations in breast cancer. In our results, amino acid sites of IRS2 mutations in CUPAx included R558P, C97Y, and A512T, of which C97Y was a new mutation not found in the public database (COSMIC). This new mutation might selectively change the function of IRS2 to promote tumor invasion, resulting in axillary lymph node metastasis presenting first. However, as our sequencing data lacked expression data, further in vivo and in vitro studies are required to verify our hypothesis and the effects of these mutations in tumor biology.

In addition, IRS2 amplification, firstly confirmed in a study of PI3K signaling pathway changes in colorectal cancer (Parsons et al. 2005), has been a vital indicator of sensitivity to targeted therapy in colorectal and lung cancers (Bertotti et al. 2015). But the drug sensitivity of IRS2 amplification in breast cancer remains unclear. A recent study established a sugar metabolism-related prognostic model that included IRS2 gene for breast cancer, which could distinguish low-risk patients who were more sensitive to chemotherapy (Mei et al. 2022). In our study, CUPAx patients with IRS2 mutations showed higher pCR rates (3/4) to neoadjuvant chemotherapy than BCAx patients (0/2) (75% vs. 0%). We speculated that IRS2 mutation might be associated with high chemotherapy sensitivity and further lead to a good prognosis in CUPAx. However, larger sample studies are warranted to verify the results.

### The discrepant pathway analysis of CUPAx and BCAx

In our data, the NOTCH pathway was less frequently and abnormally activated in the CUPAx group, which might be associated with the hidden breast lesions in CUPAx. The classical NOTCH pathway was activated during normal breast evolution and maintains mammary development (Yousefi et al. 2022; Chen et al. 2021). Abnormal activation or mutation of NOTCH genes could result in the occurrence, progression, metastasis, and treatment resistance of breast cancer (Katoh et al. 2020; Wang et al. 2015). NOTCH-3 acted as a tumor suppressor, its mutation caused tumor proliferation, invasion, and metastasis (Kontomanolis et al. 2018; Leontovich et al. 2018).

Although we have not yet found significant relationships in NOTCH pathway mutations with subtypes and prognosis, a higher amplification frequency of NOTCH3 was found in CUPAx (2/12, 16.7%) than BCAx (0/16, 0%) and TCGA-BRCA (8/401, 2.0%). NOTCH3 was reported to highly express in ER-positive and triple-negative breast cancer and contributed to prolonging OS (Zhang et al. 2021). Our results showed all CUPAx patients with NOTCH3 amplification were triple-negative subtype and survived during follow-up (Median OS: 22 months; Median DFS: 20 months vs. CUPAx without NOTCH3 amplification: Median OS: 32.8 months; Median DFS: 29.9 months, P > 0.05). Due to the limited sample size, we could not yet conclude that NOTCH amplification led to a better prognosis in CUPAx patients. However, based on the previous literature (Zhang et al. 2021), we speculated that NOTCH3 might have a certain impact on the survival of CUPAx, but further validation is needed through studies with larger sample size. It is speculative that NOTCH3 might become a vital therapeutic target for CUPAx. With over 70 clinical trials of targeting NOTCH pathway developed, some drugs like gamma-secretase inhibitors (GSI) have been extensively explored (Moore et al. 2020), verifying the therapeutic and predictive potential of NOTCH pathway genes.

Our analysis found that CUPAx had higher levels of glandular development and PML body enrichment but lower levels of positive kinase activity. Normal breast stem cells maintained normal mammary development, while genomic and epigenetic changes could turn them into breast cancer stem cells (Luo et al. 2010). No obvious primary lesions were found in the CUPAx group, suggesting that it might have more normal mammary tissues compared to BCAx. PML bodies refer to nuclear deposits of over 50 protein types, exerting essential effects in normal breast development (Plevová et al. 2007). PML acting as a tumor suppressor in vivo inhibited normal cell apoptosis and differentiation, and it could regulate tumor suppressor FOXO3 to suppress the growth of breast cancer cells (Rego et al. 2001; Sachini et al. 2019). More enriched PML body pathways in CUPAx might suggest less abnormal mammary tissues, rendering breast lesions more occult. Kinases regulated cellular biological activities through phosphorylation and various pathways activation, playing pivotal role in tumorigenesis (Cheng et al. 2011). Some kinase-related pathways, like PI3K/Akt/mTOR and MAPK, have been involved in breast cancer (García-Aranda et al. 2017). The proliferation and survival of tumor cells depended on the activity of kinases (García-Aranda et al. 2017). BCAx had a stronger regulatory effect on kinase activity, potentially fostering a more malignant phenotype in mammary tissues compared to CUPAx, thereby suggesting a poorer prognosis.

Although our sequenced sample was small-scale, we discovered some valuable mutated genes that affected vital biological functions and signaling pathways of CUPAx. We have to verify and analyze our results with a larger sample size to identify more dependable genetic or other markers that can provide a better understanding of tumor growth and development. Additionally, we hope that the potential mutations detected by F1CDx can become safer and more effective targets for CUPAx patients.

## Conclusion

CUPAx is likely to originate from the breast and shares similarities with triple-negative breast cancer. Active treatment methods such as neoadjuvant chemotherapy, mastectomy and postoperative radiotherapy could bring favorable prognosis for CUPAx. The BCAx group displayed a significantly higher mutation rate in the NOTCH pathway than the CUPAx group. Based on our enrichment analysis, we speculated this relationship to be pertinent to the concealed primary lesions of CUPAx patients.

### Supplementary Information

Below is the link to the electronic supplementary material.

Supplementary file1 Supplementary Fig. S1: Flow chart of the inclusion and exclusion (JPG 2253 KB)

Supplementary file2 Supplementary Fig. S2: The short variants (A, D, G), copy number variations (CNVs) (B, E, H), mutation types (C, F, I) in CUPAx (n = 12), BCAx (n = 16) and TCGA-BRCA (n = 401). TP53 had the highest mutation frequency among the three groups, mainly with missense mutations. Both CDK12 and ERBB2 showed amplification in CUPAx and BCAx groups. Short variants (SNVs + indels) were most frequently observed in TP53, IRS2, and PIK3CA across all three groups. Missense mutations were most prevalent in CUPAx and BCAx groups, while amplifications predominated in the TCGA-BRCA group (JPG 2557 KB)

Supplementary file3 Supplementary Fig. S3: (A) The gene mutations stratified by subtypes in CUPAx, BCAx, and TCGA-BRCA patients. The differences among three subtype groups in three groups were not significant. (B) The waterfall map of CUPAx and BCAx patients. The top three mutated genes (TP53, CDK12, ERBB2) still showed no significant differences between CUPAx and BCAx groups (JPG 5924 KB)

Supplementary file4 Supplementary Fig. S4: (A) The co-mutated analysis of CUPAx and BCAx patients. MAP3K1, BRIP1 and PIK3CA showed significant co-occurrences of mutations in the CUPAx group, while the BCAx group found significant co-occurrences of mutations in PIK3R1 with ROS1 and AXIN1. (B) The mutation sites of IRS2 and GATA6 gene in CUPAx and BCAx patients. The mutation sites of IRS2 in the CUPAx group were A512T (2/16), C97Y (1/16), and R558P (1/16). Compared with the COSMIC database, C97Y was a new site. All the GATA6 mutation sites, G244S (2/16), D5E (1/16), S184N (1/16), and H331_H333del (1/16), were novel sites (JPG 3732 KB)

Supplementary file5 (DOCX 28 KB)

## Data Availability

The raw data used to support the findings of this study are available from the corresponding author upon reasonable request.
