# Supplementary figures and images for: Clinicopathologic features and genomic profiling of female axillary lymph node metastases from adenocarcinoma or poorly differentiated carcinoma of unknown primary
Source: J Cancer Res Clin Oncol. 2024 May 15;150(5):256. doi: 10.1007/s00432-024-05783-6 (PMC11096249; doi:10.1007/s00432-024-05783-6)

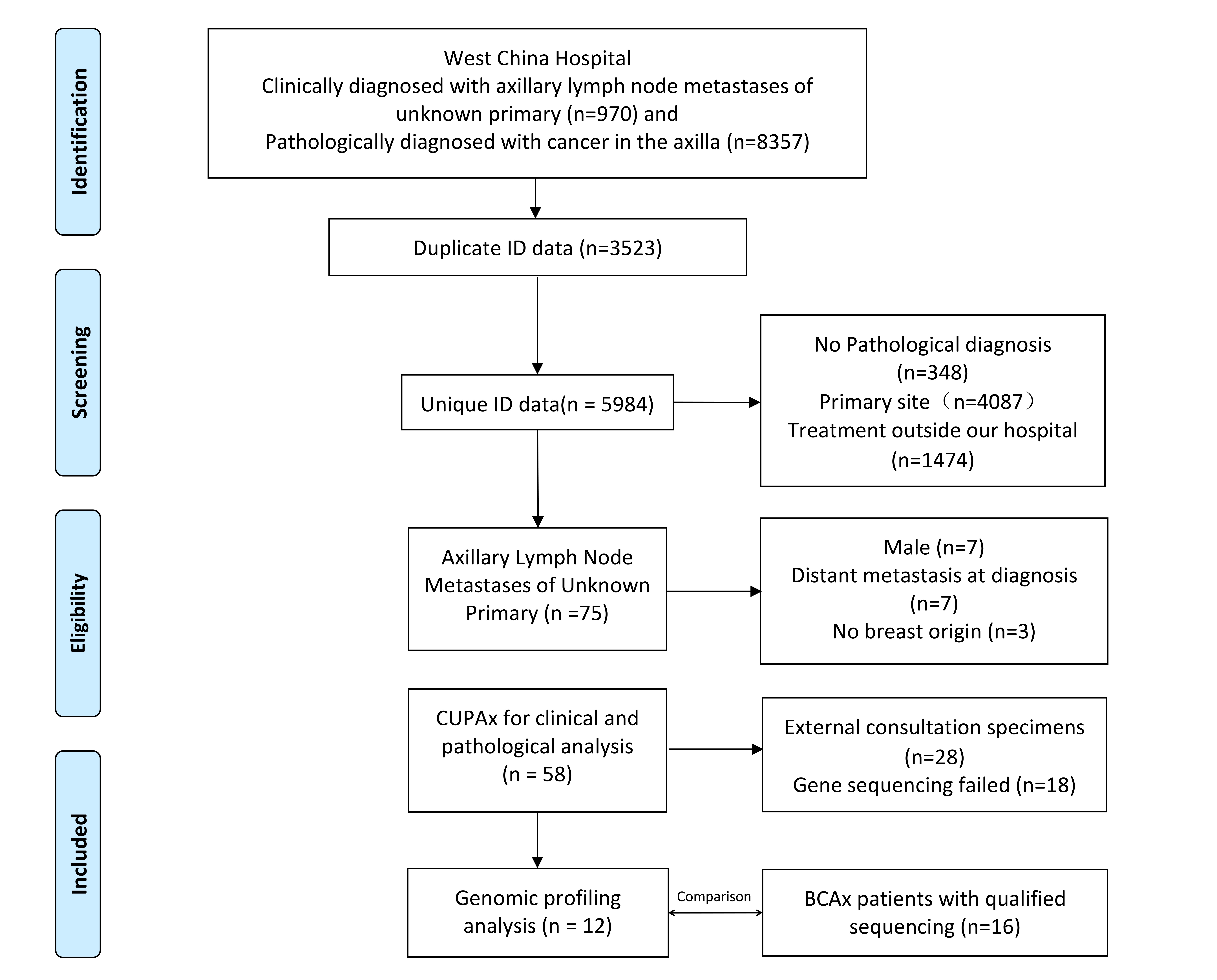

Supplement: Supplementary file 1 — Supplementary file1 Supplementary Fig. S1: Flow chart of the inclusion and exclusion (JPG 2253 KB) [file 432_2024_5783_MOESM1_ESM.jpg]

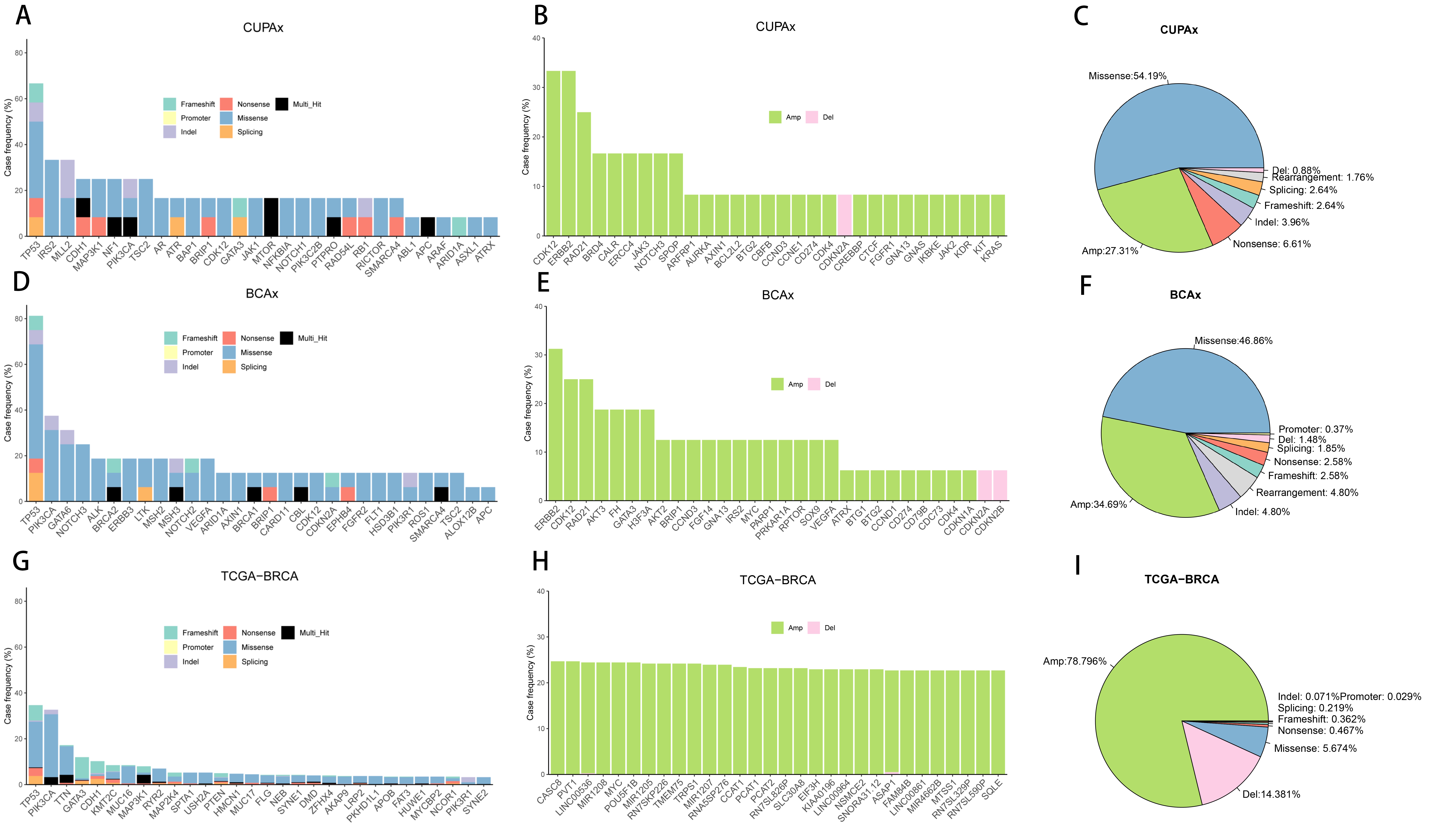

Supplement: Supplementary file 2 — Supplementary file2 Supplementary Fig. S2: The short variants (A, D, G), copy number variations (CNVs) (B, E, H), mutation types (C, F, I) in CUPAx (n = 12), BCAx (n = 16) and TCGA-BRCA (n = 401). TP53 had the highest mutation frequency among the three groups, mainly with missense mutations. Both CDK12 and ERBB2 showed amplification in CUPAx and BCAx groups. Short variants (SNVs + indels) were most frequently observed in TP53, IRS2, and PIK3CA across all three groups. Missense mutations were most prevalent in CUPAx and BCAx groups, while amplifications predominated in the TCGA-BRCA group (JPG 2557 KB) [file 432_2024_5783_MOESM2_ESM.jpg]
