# Supplementary material for: Clinicopathologic features and genomic profiling of female axillary lymph node metastases from adenocarcinoma or poorly differentiated carcinoma of unknown primary
Source: J Cancer Res Clin Oncol. 2024 May 15;150(5):256. doi: 10.1007/s00432-024-05783-6 (PMC11096249; doi:10.1007/s00432-024-05783-6)
Supplement: Supplementary file 5 — Supplementary file5 (DOCX 28 KB) [file 432_2024_5783_MOESM5_ESM.docx]

**Supplementary Table S1**. Baseline characteristics of 58 CUPAx in West China Hospital.

| **Variables** | **Mean+SD/ N (%)** | **Variables** | **Mean+SD/ N (%)** |
| --- | --- | --- | --- |
| **Ki67** | 40.98 ± 22.11 | **Surgery** |  |
| **Age group** |  | Mastectomy | 35 (60.34%) |
| <55 | 38 (65.52%) | BCS | 8 (13.79%) |
| >=55 | 20 (34.48%) | ALND | 4 (6.90%) |
| **Year group** |  | ALNS | 11 (18.97%) |
| 2009-2014 | 14 (24.14%) | **Breast Operation** |  |
| 2015-2021 | 44 (75.86%) | No | 20 (34.48%) |
| **Location** |  | Mastectomy | 35 (60.34%) |
| Left | 41 (70.69%) | Unknown | 3 (5.17%) |
| Right | 17 (29.31%) | **Axillary Operation** |  |
| **N stage** |  | ALND | 38 (65.52%) |
| cN1 | 28 (48.28%) | Excision | 8 (13.79%) |
| cN2 | 9 (15.52%) | ALNS | 12 (20.69%) |
| cN3 | 21 (36.21%) | **Radiotherapy** |  |
| **ER** |  | No | 18 (31.03%) |
| Negative | 31 (53.45%) | Chest wall/breast +SCF/IVF | 21 (36.21%) |
| Positive | 22 (37.93%) | Chest wall/breast +axilla +SCF/IVF | 4 (6.90%) |
| Unknown | 5 (8.62%) | Axilla+SCF/IVF | 1 (1.72%) |
| **PR** |  | SCF/IVF | 1 (1.72%) |
| Negative | 33 (56.90%) | No specific region | 7(12.07%) |
| Positive | 19 (32.76%) | Unknown | 6 (10.34%) |
| Unknown | 6 (10.34%) | **Chemotherapy** |  |
| **HER2** |  | No | 7 (12.07%) |
| Negative | 36 (62.07%) | Yes | 48 (82.76%) |
| Positive | 15 (25.86%) | Unknown | 3 (5.17%) |
| Unknown | 7 (12.07%) | **Chemotherapy type** |  |
| **Subtype** |  | Neoadjuvant | 12 (20.69%) |
| HR+/HER2+ | 10 (17.24%) | Neoadjuvant  + adjuvant | 16 (27.59%) |
| HR+/HER2- | 15 (25.86%) | Adjuvant | 19 (32.76%) |
| HR-/HER2+ | 5 (8.62%) | NA | 11 (18.97%) |
| HR-/HER2- | 22 (37.93%) | **Endocrine therapy** |  |
| Unknown | 6 (10.34%) | No | 32 (55.17%) |
| **Axillary Downstaging(pCR/ypN+)** |  | Yes | 20 (34.48%) |
| pCR | 12 (20.69%) | Unknown | 6 (10.34%) |
| ypN+ | 13 (22.41%) | **Target therapy** |  |
| Unknown | 33 (56.90%) | No | 39 (67.24%) |
| **Positive axillary lymph node ratio** |  | Yes | 15 (25.86%) |
| <25% | 30 (51.72%) | Unknown | 4 (6.90%) |
| >=25% | 28 (48.28%) | **Primary breast site** |  |
| **Pathology** |  | No | 52 (89.66%) |
| Invasive carcinoma of no special type | 25 (43.10%) | Yes | 6 (10.34%) |
| DC | 1 (1.72%) | **Chemotherapy regimen** |  |
| IDC | 5 (8.62%) | Anthracycline-based | 20 (34.48%) |
| ILC | 1 (1.72%) | Paclitaxel-platinum | 20 (34.48%) |
| PDC | 7 (12.07%) | Other | 7 (12.07%) |
| Unclassified/Unknown | 19 (32.76%) | Unknown/No | 11 (18.97%) |
| **Breast Biopsy** |  |  |  |
| No | 43 (74.14%) |  |  |
| Yes | 15 (25.86%) |  |  |

DC: [ductal carcinoma](javascript:;), IDC: invasive [ductal carcinoma](javascript:;), ILC: invasive lobular carcinoma, PDC: [poorly differentiated carcinoma](javascript:;). One patient received chemotherapy of an unspecified type and was classified as NA.

**Supplementary Table S2**. Univariable analysis of OS and DFS in CUPAx patients.

| **Variables** | **OS** | **DFS** |
| --- | --- | --- |
| **Age** |  |  |
| <=55 | 1 | 1 |
| >55 | 1.18 (0.29, 4.76) 0.82 | 1.73 (0.39, 7.73) 0.47 |
| N **stage** |  |  |
| cN1 | 1 | 1 |
| cN2 | 0.67 (0.07, 6.08) 0.72 | 0.72 (0.08, 6.46) 0.77 |
| cN3 | 1.82 (0.45, 7.37) 0.40 | 0.74 (0.13, 4.06) 0.73 |
| **Location** |  |  |
| Left | 1 | 1 |
| Right | 1.22 (0.30, 4.91) 0.78 | 0.44 (0.05, 3.70) 0.45 |
| **Subtype** |  |  |
| HR+/HER2+ | 1 | 1 |
| HR+/HER2- | § | § |
| HR-/HER2+ | § | § |
| HR-/HER2- | § | § |
| Unknown | § | § |
| **Surgery** |  |  |
| Mastectomy | 1 | 1 |
| BCS | § | 2.97 (0.32, 27.11) 0.34 |
| ALND | § | 4.16 (0.42, 41.15) 0.22 |
| ALNS | § | 1.91 (0.21, 17.31) 0.56 |
| **Breast Operation** |  |  |
| No | 1 | 1 |
| Mastectomy | § | 0.37 (0.08, 1.71) 0.20 |
| Unknown | § | § |
| **Axillary Operation** |  |  |
| ALND | 1 | 1 |
| Excision | 7.09 (1.18, 42.54) **0.03** | 3.49 (0.64, 19.07) 0.15 |
| ALNS | 16.40 (2.79, 96.48) **<0.01** | 1.88 (0.21, 16.88) 0.57 |
| **Axillary Downstaging(pCR/ypN+)** |  |  |
| pCR | 1 | 1 |
| ypN+ | § | § |
| Unknown | § | § |
| **Positive axillary lymph node ratio** |  |  |
| <25% | 1 | 1 |
| >=25% | 14.28 (1.77, 114.98) **0.01** | 4.89 (0.94, 25.43) 0.06 |
| **Radiotherapy** |  |  |
| No | 1 | 1 |
| Yes | 0.06 (0.01, 0.57) **0.01** | 0.30 (0.07, 1.36) 0.12 |
| Unknown | 5.09 (0.91, 28.60) 0.06 | § |
| **Radiotherapy sites** |  |  |
| Chest wall/breast+SCF/IVF | 1 | 1 |
| Chest wall/breast+axilla+SCF/IVF | § | 5.87 (0.37, 94.25) 0.21 |
| Axilla+SCF/IVF | 1 | § |
| SCF/IVF | § | 38.80 (1.69, 891.87) **0.02** |
| **Chemotherapy** |  |  |
| No | 1 | 1 |
| Yes | 0.29 (0.05, 1.60) 0.16 | 0.28 (0.05, 1.43) 0.13 |
| Unknown | 3.07 (0.27, 34.98) 0.37 | § |
| **Chemotherapy regimen** |  |  |
| Anthracycline-based | 1 | 1 |
| Paclitaxel-platinum | § | 1.43 (0.13, 15.73) 0.77 |
| Other | § | 1.56 (0.14, 17.24) 0.72 |
| Unknown/No | § | 4.90 (0.44, 54.20) 0.19 |
| **Endocrine therapy** |  |  |
| No | 1 | 1 |
| Yes | 0.27 (0.05, 1.53) 0.14 | 1.52 (0.34, 6.84) 0.58 |
| Unknown | 3.86 (0.72, 20.74) 0.11 | § |
| **Target therapy** |  |  |
| No | 1 | 1 |
| Yes | 0.41 (0.05, 3.39) 0.41 | 0.89 (0.17, 4.60) 0.89 |
| Unknown | 10.36 (1.83, 58.60) 0.01 | § |

N=54, §: The model failed because of the small sample size. OS, overall survival; DFS, disease-free survival. Not all patients were reported DFS and OS since in some subgroups, there were insufficient samples for effective statistical analysis.

**Supplementary Table S3.** Multivariate analysis of OS in CUPAx patients. The variables included were Radiotherapy, Positive axillary lymph node ratio, and Axillary Operation.

| **Variables** | **OS**  **HR (95%CI) P value** |
| --- | --- |
| **Radiotherapy** |  |
| No | 1 |
| Yes | 0.05 (0.00, 0.93) **0.04** |
| **Positive axillary lymph node ratio** |  |
| <25% | 1 |
| >=25% | 8.98 (0.73, 110.03) 0.09 |
| **Axillary Operation** |  |
| ALND | 1 |
| Excision | 1.13 (0.14, 8.97) 0.91 |
| ALNS | 2.20 (0.26, 18.78) 0.47 |

N=52 (excluded patients with unknown variables). ALND: axillary lymph node dissection, ALNS: axillary lymph node sampling, OS, overall survival.

**Supplementary Table S4**. Baseline characteristics of 12 CUPAx and 16 BCAx patients who received comprehensive genomic profiling in West China Hospital.

| **Group** | **CUPAx** | **BCAx** | **P value** |
| --- | --- | --- | --- |
| **N** | 12 | 16 |  |
| **Age** |  |  | 0.18 |
| <=55 | 9 (75.00%) | 8 (50.00%) |  |
| >55 | 3 (25.00%) | 8 (50.00%) |  |
| **Location** |  |  | 0.31 |
| Left | 9 (75.00%) | 9 (56.25%) |  |
| Right | 3 (25.00%) | 7 (43.75%) |  |
| **Sample type** |  |  | 0.35 |
| Excision/Surgery | 5 (41.67%) | 4 (25.00%) |  |
| Sampling | 7 (58.33%) | 12 (75.00%) |  |
| N **stage** |  |  | 0.44 |
| cN1 | 6 (50.00%) | 5 (31.25%) |  |
| cN2 | 2 (16.67%) | 6 (37.50%) |  |
| cN3 | 4 (33.33%) | 5 (31.25%) |  |
| **ER** |  |  | 0.66 |
| Negative | 5 (41.67%) | 8 (50.00%) |  |
| Positive | 7 (58.33%) | 8 (50.00%) |  |
| **PR** |  |  | 0.74 |
| Negative | 6 (50.00%) | 9 (56.25%) |  |
| Positive | 6 (50.00%) | 7 (43.75%) |  |
| **HER2** |  |  | **0.02** |
| Negative | 10 (83.33%) | 6 (37.50%) |  |
| Positive | 2 (16.67%) | 10 (62.50%) |  |
| **Subtype** |  |  | 0.09 |
| HR+/HER2+ | 1 (8.33%) | 5 (31.25%) |  |
| HR+/HER2- | 7 (58.33%) | 3 (18.75%) |  |
| HR-/HER2+ | 1 (8.33%) | 5 (31.25%) |  |
| HR-/HER2- | 3 (25.00%) | 3 (18.75%) |  |
| **Ki67** | 43.18 ± 24.73 | 40.94 ± 16.35 | 0.78 |
| **Surgery** |  |  | **0.02** |
| Mastectomy | 6 (50.00%) | 16 (100.00%) |  |
| BCS | 4 (33.33%) | 0 (0.00%) |  |
| ALND | 1 (8.33%) | 0 (0.00%) |  |
| ALNS | 1 (8.33%) | 0 (0.00%) |  |
| **Breast Operation** |  |  | **<0.01** |
| No | 6 (50.00%) | 0 (0.00%) |  |
| Mastectomy | 6 (50.00%) | 16 (100.00%) |  |
| **Axillary Operation** |  |  | 0.16 |
| ALND | 8 (66.67%) | 15 (93.75%) |  |
| ALNS | 1 (8.33%) | 0 (0.00%) |  |
| Excision | 3 (25.00%) | 1 (6.25%) |  |
| **Positive axillary lymph node ratio** |  |  | 0.14 |
| <=25 | 3 (25.00%) | 8 (53.33%) |  |
| >25 | 9 (75.00%) | 7 (46.67%) |  |
| **Axillary Downstaging(pCR/ypN+)** |  |  | **0.04** |
| Unknown | 4 (33.33%) | 5 (31.25%) |  |
| pCR | 7 (58.33%) | 3 (18.75%) |  |
| ypN+ | 1 (8.33%) | 8 (50.00%) |  |
| **Radiotherapy** |  |  | 0.17 |
| No | 6 (50.00%) | 4 (25.00%) |  |
| Yes | 6 (50.00%) | 12 (75.00%) |  |
| **Chemotherapy** |  |  | 0.83 |
| No | 1 (8.33%) | 1 (6.25%) |  |
| Yes | 11 (91.67%) | 15 (93.75%) |  |
| **Neoadjuvant chemotherapy** |  |  | 0.71 |
| No | 3 (25.00%) | 5 (31.25%) |  |
| Yes | 9 (75.00%) | 11 (68.75%) |  |
| **Endocrine therapy** |  |  | 0.58 |
| No | 8 (66.67%) | 9 (56.25%) |  |
| Yes | 4 (33.33%) | 7 (43.75%) |  |
| **Target therapy** |  |  | 0.38 |
| No | 8 (66.67%) | 8 (50.00%) |  |
| Yes | 4 (33.33%) | 8 (50.00%) |  |
| **OS** |  |  | 0.83 |
| Alive | 11 (91.67%) | 15 (93.75%) |  |
| Dead | 1 (8.33%) | 1 (6.25%) |  |
| **OS Follow-up(months)** | 31.00 ± 18.21 | 31.25 ± 22.28 | 0.98 |
| **Reccurence/Metastasis** |  |  | 0.18 |
| No | 8 (66.67%) | 14 (87.50%) |  |
| Yes | 4 (33.33%) | 2 (12.50%) |  |
| **DFS Follow-up(months)** | 28.25 ± 20.12 | 29.69 ± 22.52 | 0.86 |

BCS: breast-conseving surgery, ALND: axillary lymph node dissection, ALNS: axillary lymph node sampling. OS, overall survival; DFS, disease-free survival.

**Supplementary Table S5.** The clinically relevant gene mutation sites and types of 12 CUPAx and 16 BCAx patients.

| **ID** | **Year** | **Age** | **Group** | **TMB** | **MSI** | **Gene mutations** |
| --- | --- | --- | --- | --- | --- | --- |
| 11090 | 2021 | 41 | CUPAx | 3 | MSS | BRCA2 - splice site 9117G>A |
| 11088 | 2021 | 55 | CUPAx | 3 | MSS | None |
| 11089 | 2020 | 48 | CUPAx | 1 | MSS | ERBB2 - amplification |
| 11093 | 2020 | 64 | CUPAx | 6 | MSS | PIK3CA - E545K |
| 11096 | 2016 | 56 | CUPAx | 1 | MSS | ERBB2 - amplification |
| 14958 | 2020 | 53 | CUPAx | 13 | MSS | BRIP1 - E910* |
| 14980 | 2020 | 72 | CUPAx | 30 | MSS | CDK4 - amplification;NF1 - E524*, R416* - subclonal; PIK3CA - H1047R, amplification |
| 14981 | 2018 | 46 | CUPAx | 0 | MSS | None |
| 14983 | 2018 | 52 | CUPAx | 8 | MSS | TSC2 - loss exons 5-42 |
| 14984 | 2021 | 55 | CUPAx | 0 | MSS | None |
| 14992 | 2016 | 50 | CUPAx | NA | MSS | ERBB2 - amplification |
| 14999 | 2020 | 74 | CUPAx | 5 | MSS | KIT - amplification - equivocal; PDGFRA - amplification - equivocal |
| 11081 | 2020 | 58 | BCAx | NA | MSS | ERBB2 - amplification |
| 11082 | 2020 | 59 | BCAx | 11 | MSS | BRCA2 - S2267*, T302fs*21;BRIP1 - E454* |
| 11074 | 2021 | 57 | BCAx | 3 | MSS | PIK3CA - K111N |
| 11075 | 2021 | 43 | BCAx | 4 | MSS | ERBB2 - amplification |
| 11076 | 2021 | 68 | BCAx | 4 | MSS | PIK3CA - E545K |
| 11077 | 2021 | 79 | BCAx | 5 | MSS | AKT2 - amplification; MYC - amplification |
| 11083 | 2020 | 35 | BCAx | 11 | MSS | ERBB3 - E928G; PIK3CA - D1017H |
| 11084 | 2020 | 46 | BCAx | 1 | MSS | CCND1 - amplification |
| 11087 | 2019 | 51 | BCAx | 8 | MSS | BRCA2 - V220fs*4; CDK4 - amplification; |
| 14952 | 2020 | 63 | BCAx | 4 | MSS | PIK3CA - P104_V105del |
| 14953 | 2021 | 44 | BCAx | 1 | MSS | ERBB2 - amplification |
| 14954 | 2021 | 58 | BCAx | 3 | MSS | NF1 - loss exons 1-36 |
| 14987 | 2018 | 41 | BCAx | NA | MSS | CD274 (PD-L1) - amplification; PDCD1LG2 (PD-L2) - amplification |
| 14988 | 2019 | 58 | BCAx | NA | MSS | AKT3 - amplification; ERBB2 - amplification |
| 14990 | 2019 | 52 | BCAx | 6 | MSS | BRCA1 - D1692N; PIK3CA - Q546K |
| 14991 | 2019 | 52 | BCAx | 0 | MSS | ERBB2 - amplification; PIK3CA - E542K |

TMB, tumor mutation burden, MSI: microsatellite instability.
